# Supplementary material for: Combined Association of Plasma Metabolites with Body Mass Index and Physical Activity Level
Source: Biology (Basel). 2024 Dec 20;13(12):1074. doi: 10.3390/biology13121074 (PMC11673513; doi:10.3390/biology13121074)
Supplement: Supplementary file 1 [file biology-13-01074-s001.zip › biology-3068015-supplementary.pdf]

Supplementary Material

Combined Association of Plasma Metabolites with Body Mass Index and Physical Activity Level

Mayara Lambert [1], Larissa de Castro Pedroso[1], Álex Aparecido Rosini Silva[2], Leonardo Henrique Dalcheco Messias[1], Andréia M. Porcari[2], Patrícia de Oliveira Carvalho[2], Pedro Paulo de Menezes Scariot and Ivan Gustavo Masselli dos Reis[1,*]

[1] Research Group on Technology Applied to Exercise Physiology - GTAFE, Health Sciences Postgraduate Program, São Francisco University, Bragança Paulista 12916-900, SP, Brazil; lambert.mayara@mail.usf.edu.br (M.L.); larissa.pedroso@mail.usf.edu.br (L.C.P.); leonardo.messias@usf.edu.br (L.H.D.M.); pedro.scariot@mail.usf.edu.br (P.P.M.S.)
[2] MS4Life Laboratory of Mass Spectrometry, Health Sciences Postgraduate Program, São Francisco University, Bragança Paulista 12916-900, SP, Brazil; alex.rosini@mail.usf.edu.br (A.A.R.S.); andreia.porcari@usf.edu.br (A.M.P.); patricia.carvalho@usf.edu.br (P.O.C.)
* Correspondence: ivan.reis@usf.edu.br

Information regarding all metabolites present in the network model can be found in Tables S1 (identified) and S2 (undentified), including eigenvector centrality rank, metabolite notations, adducts, chemical formula, mass error, retention tine, ANOVA F-values (F.val), main effects (p), efect size (E.S.), statistical power (Power).

Table S1. Identified Compounds

Eigenvector Rank	Metabolite	Adducts	Formula	Mass Error (ppm)	m/z measured	Retention time (min)	BMI (F.val)	IPAQ (F.val)	Interaction (F.val)	BMI (p)	IPAQ (p)	Interaction (p)	BMI (E.S.)	IPAQ (E.S.)	BMI (Power)	IPAQ (Power)
1st	PC 36:4;O2	M+FA-H	C44H80NO9P	2.15	842.557	3.15	7.5	2.842	3.882	0.009	0.098	0.054	0.82	0.43	0.99	0.83
2nd	PC 20:4;O/16:0	M+FA-H	C44H80NO9P	2.12	842.557	2.78	7.4	2.550	4.366	0.009	0.116	0.042	0.83	0.38	0.99	0.75
3rd	PE 20:3;O/18:0	M-H2O-H. M-H	C43H78NO9P	1.66	782.5345	3.39	9.2	2.326	6.202	0.004	0.133	0.016	0.83	0.33	0.99	0.62
4th	PE 20:2/PGE1	M+FA-H	C45H80NO11P	0.75	886.5457	2.65	7.2	1.857	4.449	0.010	0.179	0.040	0.75	0.27	0.99	0.46
5th	PS 22:4/PGE1	M+FA-H	C48H80NO13P	-2.05	954.5331	2.65	9.2	1.696	4.148	0.004	0.199	0.047	0.88	0.23	0.99	0.36
6th	PE 20:2/PGE1	M+FA-H	C45H80NO11P	2.96	886.5476	2.85	7.7	1.544	4.982	0.008	0.220	0.030	0.80	0.21	0.99	0.30
7th	Prostaglandin D1	M-H2O-H	C20H34O5	-2.34	335.222	0.42	9.6	2.393	4.523	0.003	0.128	0.038	0.83	0.34	0.99	0.65

Eigenvector Rank	Metabolite	Adducts	Formula	Mass Error (ppm)	m/z measured	Retention time (min)	BMI (F.val)	IPAQ (F.val)	Interaction (F.val)	BMI (p)	IPAQ (p)	Interaction (p)	BMI (E.S.)	IPAQ (E.S.)	BMI (Power)	IPAQ (Power)
8th	PG 40:5;O2	M+FA-H	C46H81O12P	3.17	901.5475	3.75	10.9	4.902	4.459	0.002	0.031	0.040	0.90	0.57	0.99	0.98
9th	PI 20:3;O/18:1	M-H	C47H83O14P	3.18	901.5476	3.46	9.5	4.629	3.287	0.003	0.036	0.076	0.87	0.56	0.99	0.97
10th	PS 18:0/20:4	M-H	C45H80NO9P	0.55	808.5502	4.28	8.8	1.926	4.827	0.005	0.171	0.033	0.88	0.40	0.99	0.79
11th	PE 20:4;O/18:0	M-H	C43H78NO9P	1.99	782.5357	2.78	7.7	2.301	5.566	0.008	0.135	0.022	0.79	0.29	0.99	0.52
12th	PE 22:2/PGE1	M+FA-H	C47H84NO11P	1.13	914.5774	3.57	9.2	2.237	4.525	0.004	0.141	0.038	0.98	0.28	0.99	0.48
13th	PC 16:0/20:4;O	M+FA-H	C44H80NO9P	2.59	842.5573	3.39	6.9	2.330	4.098	0.011	0.133	0.048	0.77	0.38	0.99	0.74
14th	PS 22:4/PGE1	M+FA-H	C48H80NO13P	-1.75	954.5333	2.85	7.7	2.227	4.802	0.008	0.142	0.033	0.79	0.30	0.99	0.53
15th	PC 18:1;O/18:0	M+FA-H	C44H84NO9P	2.92	846.5889	3.57	9.1	1.586	3.960	0.004	0.214	0.052	0.97	0.23	0.99	0.34
16th	PC 18:1;O/18:0	M+FA-H	C44H84NO9P	2.73	846.5888	3.73	8.9	1.812	3.744	0.004	0.184	0.059	0.99	0.22	0.99	0.32
18th	PC 20:4;O/18:0	M+FA-H	C46H84NO9P	2.58	870.5887	3.67	8.5	2.320	5.070	0.005	0.134	0.029	0.90	0.36	0.99	0.70
19th	PE 18:1;O/20:0	M-H	C43H82NO9P	3.16	786.5679	3.57	11.2	1.677	4.280	0.002	0.201	0.044	1.08	0.21	1	0.29
20th	PE 18:1;O2/18:0	M-H2O-H	C41H80NO10P	1.92	758.5356	2.65	9.1	1.756	4.070	0.004	0.191	0.049	0.85	0.21	0.99	0.30
21st	PC 18:1;O/16:0	M+FA-H	C42H80NO9P	3.33	818.5578	2.27	6.5	1.485	4.217	0.014	0.229	0.045	0.74	0.16	0.99	0.19
22nd	PE 18:1;O/18:0	M-H2O-H	C41H80NO10P	2.17	758.5358	2.85	8.1	2.174	3.976	0.006	0.146	0.052	0.81	0.25	0.99	0.40
23rd	PC 18:1;O/16:0	M+FA-H	C42H80NO9P	2.15	818.5569	2.65	8.9	1.990	3.738	0.004	0.164	0.059	0.82	0.23	0.99	0.34
24th	PC 18:1;O/16:0	M+FA-H	C42H80NO9P	2.19	818.557	2.85	8.5	1.796	3.616	0.005	0.186	0.063	0.81	0.22	0.99	0.31
25th	Prostaglandin D2	M-H	C20H32O5	-1.22	351.2173	0.4	12.2	5.561	7.504	0.001	0.022	0.008	0.81	0.49	0.99	0.91
26th	PC 20:3;O2/18:1	M+FA-H	C46H84NO9P	0.87	870.5873	4.33	9.2	2.273	4.724	0.004	0.138	0.034	0.91	0.36	0.99	0.70
27th	PC 20:3;O/18:0	M+FA-H	C46H84NO9P	3.1	870.5891	4	8.5	2.635	4.777	0.005	0.111	0.033	0.92	0.41	0.99	0.81
28th	PC 18:1;O/20:4	M+FA-H	C46H80NO9P	2.99	866.5577	2.64	9.0	2.959	3.898	0.004	0.091	0.054	0.95	0.43	0.99	0.84
29th	Epoxyeicosatrienoic acid	M-H	C20H32O3	-2.05	319.2272	0.59	7.0	3.244	3.373	0.011	0.078	0.072	0.80	0.52	0.99	0.94
30th	PC 18:1;O2/18:3	M+FA-H	C44H80NO10P	2.94	858.5526	1.56	6.5	2.045	4.915	0.014	0.159	0.031	0.51	0.32	0.94	0.60
32nd	PC 20:4/18:1;O	M+FA-H	C46H80NO9P	3.09	866.5578	3.29	8.5	2.442	4.967	0.005	0.124	0.030	0.91	0.36	0.99	0.68
33rd	Am-Hex-PE O-36:5	M-H2O-H	C47H84NO12P	3.22	866.5581	3.06	10.3	2.628	3.403	0.002	0.111	0.071	1.01	0.38	0.99	0.75
35th	PC 20:1;O	M-H2O-H	C28H54NO9P	-0.02	560.3358	0.65	15.2	1.243	3.702	0.000	0.270	0.060	1.09	0.24	1	0.37
36th	PE 38:4;O	M-H	C43H78NO9P	2.98	782.5365	4.1	11.5	2.296	2.830	0.001	0.136	0.099	0.67	0.31	0.99	0.56

Eigenvector Rank	Metabolite	Adducts	Formula	Mass Error (ppm)	m/z measured	Retention time (min)	BMI (F.val)	IPAQ (F.val)	Interaction (F.val)	BMI (p)	IPAQ (p)	Interaction (p)	BMI (E.S.)	IPAQ (E.S.)	BMI (Power)	IPAQ (Power)
37th	DG 42:5;O2	M+Na-2H	C45H78O8	-2.17	767.5427	4.28	14.8	3.073	5.965	0.000	0.086	0.018	0.96	0.35	0.99	0.66
38th	FA 22:5;O2	M-H2O-H	C22H34O4	-1.63	343.2273	0.5	8.2	3.476	1.410	0.006	0.068	0.241	0.93	0.57	0.99	0.97
39th	PC 22:4/18:1;O	M+FA-H	C48H84NO9P	2.47	894.5887	3.57	10.8	3.382	3.074	0.002	0.072	0.086	1.08	0.43	1	0.83
40th	PC 18:1;O/18:2	M+FA-H	C44H80NO9P	2.15	842.557	1.96	8.3	2.079	4.762	0.006	0.155	0.034	0.82	0.13	0.99	0.15
41st	PS 20:3/22:0	M-H2O-H	C48H88NO10P	2.85	850.5992	7.43	10.6	3.049	2.527	0.002	0.087	0.118	0.59	0.37	0.98	0.72
42nd	PS 16:0/20:1	M+FA-H	C42H80NO10P	0.81	834.5508	1.79	9.0	1.379	2.241	0.004	0.246	0.141	0.62	0.093	0.99	0.10
43rd	MG 0:0/18:3/0:0	2M-H	C21H36O4	-0.29	703.5152	4.31	12.6	1.129	2.048	0.001	0.293	0.159	1.07	0.31	1	0.58
45th	PG 38:3;O2	M+FA-H	C44H81O12P	3.24	877.5475	3.24	12.8	0.673	1.800	0.001	0.416	0.186	0.85	0.31	0.99	0.58
47th	PC 20:4;O/20:0	M-H2O-H	C48H88NO9P	2.22	834.6037	8.38	10.9	2.464	3.730	0.002	0.123	0.059	0.82	0.25	0.99	0.39
48th	PE 20:1/PGJ2	M-H2O-H	C45H78NO10P	3.56	804.5214	4.47	8.1	0.768	0.085	0.006	0.385	0.772	0.50	0.46	0.93	0.88
49th	PC 20:4/18:1	M-H	C46H82NO8P	2.54	806.5726	6.34	9.8	0.882	3.908	0.003	0.352	0.053	0.80	0.13	0.99	0.15
50th	PC 40:7;O	M-H	C48H82NO9P	3.3	846.5682	5.21	9.3	2.152	0.021	0.004	0.149	0.885	0.48	0.49	0.90	0.92
52nd	PE 20:0	M-H	C25H50NO8P	2.22	522.3213	0.98	7.7	2.620	2.386	0.008	0.112	0.129	0.42	0.51	0.82	0.93
53rd	PE 38:3;O	M-H	C43H80NO9P	3.02	784.5522	6.63	6.7	1.979	0.091	0.012	0.166	0.764	0.54	0.46	0.96	0.88
54th	PE 20:4/18:2	M-H. M+Na-2H	C43H74NO8P	3.96	784.5452	4.31	11.1	0.842	2.802	0.002	0.363	0.100	0.87	0.20	0.99	0.27
55th	PE 20:3;O/16:0	M-H	C41H76NO9P	2.51	756.5204	5.03	8.0	3.351	0.299	0.007	0.073	0.587	0.47	0.44	0.90	0.85
56th	PS 18:0;O/20:0	M+Na-2H	C44H88NO9P	3.73	826.5973	7.83	8.7	1.024	1.469	0.005	0.316	0.231	0.47	0.44	0.90	0.85
58th	PE 20:0/PGJ2	M-H2O-H	C45H80NO10P	-0.48	806.5337	4.87	13.4	2.675	2.112	0.001	0.108	0.152	0.58	0.37	0.98	0.71
59th	PC 18:1;O/16:1	M-H2O-H	C42H78NO9P	1.45	752.5247	4.33	10.7	0.307	5.727	0.002	0.582	0.020	0.76	0.12	0.99	0.13
60th	PE 20:1;O	M-H2O-H	C25H48NO9P	0.41	518.289	0.56	6.3	0.211	3.030	0.015	0.648	0.088	0.51	0.27	0.51	0.46
62nd	DG 32:1	M+FA-H	C35H66O5	2.83	611.4908	1.3	9.4	1.267	2.984	0.004	0.266	0.090	0.72	0.080	0.99	0.09
63rd	FAHFA 34:4	M-H	C34H58O4	0.58	529.4265	3.22	7.5	0.096	2.174	0.008	0.758	0.146	0.72	0.289	0.99	0.51

Effect size (ES) can be trivial (< 0.19), small (0.2–0.59), moderate (0.6–1.19), large (1.2–1.99), and very large (> 2.0); Statistical power can be low (< 0.29), moderate (0.3-0.79), high (0.8 >);

DG: Diacylglycerol; FA: Docosanoid; FAHFA: Fatty Acyl esters of Hydroxy Fatty Acids; MG: Monoacylglycerol; PC: Phosphatidylcholine; PE: Phosphatidylethanolamine; PS: Phosphatidylserine; PG Phosphatidylglycerol; PI: Phosphatidylinositol

Table S2. Unidentified Compounds

Eigenvector Rank	Retention time (min)	m/z measured	BMI (F.val)	IPAQ (F.val)	Interaction (F.val)	BMI (p)	IPAQ (p)	Interaction (p)	BMI (E.S.)	IPAQ (E.S.)	BMI (Power)	IPAQ (Power)
17th	3.2	901.5467	9.7	5.250	3.311	0.003	0.026	0.075	0.85	0.63	0.99	0.99
31st	0.38	411.1968	6.6	3.556	4.187	0.013	0.065	0.046	0.59	0.39	0.98	0.77
34th	4.28	834.5344	12.7	0.814	3.633	0.001	0.371	0.062	0.99	0.33	0.99	0.61
44th	0.56	321.2386	7.2	7.732	1.095	0.010	0.008	0.300	0.77	0.70	0.99	0.99
46th	0.86	562.3523	7.8	2.259	3.926	0.007	0.139	0.053	0.75	0.29	0.99	0.51
51st	0.68	494.2889	8.5	2.740	2.194	0.005	0.104	0.145	0.38	0.51	0.74	0.94
57th	6.34	808.5466	8.2	1.557	2.124	0.006	0.218	0.151	0.52	0.44	0.95	0.86
61st	3.59	792.4982	8.1	0.004	0.087	0.006	0.952	0.770	0.76	0.074	0.99	0.08
64th	9.63	816.6228	7.6	0.222	4.784	0.008	0.640	0.033	0.69	0.29	0.99	0.50

Effect size (ES) can be trivial (< 0.19), small (0.2–0.59), moderate (0.6–1.19), large (1.2–1.99), and very large (> 2.0); Statistical power can be low (< 0.29), moderate (0.3-0.79), high (0.8 >);

DG: Diacylglycerol; FA: Docosanoid; FAHFA: Fatty Acyl esters of Hydroxy Fatty Acids; MG: Monoacylglycerol; PC: Phosphatidylcholine; PE: Phosphatidylethanolamine; PS: Phosphatidylserine; PG Phosphatidylglycerol; PI: Phosphatidylinositol

Pearson coeficients of correlation between BMI, IPAQ and the 25 highest eigenvector centralities can be found in the Table S3.

Table S3. Coefficients of Pearson correlation among body mass index (BMI), index of physical activity questionnaire (IPAQ) and the plasma metabolites in the top 25 eigenvector rank of centralities

	Eigenvector Rank		1st	2nd	3rd	4th	5th	6th	7th	8th	9th	10th	11th	12th	13th	14th	15th	16th	17th	18th	19th	20th	21st	22nd	23rd	24th	25th
	BMI	IPAQ	PC	PC	PE	PE	PS	PE	PGD1	PG	PI	PS	PE	PE	PC	PS	PC	PC	3.2 901.5m/z	PC	PE	PE	PC	PE	PC	PC	PGD2
BMI		-0,20	-0,42	-0,42	-0,43	0,44	0,45	0,45	0,29	-0,55	0,48	0,22	-0,35	0,47	-0,43	0,45	-0,46	-0,44	0,48	-0,41	0,45	0,48	-0,47	0,49	-0,40	0,49	-0,06
IPAQ	-0,20		0,29	0,30	0,12	-0,31	-0,31	-0,25	-0,20	0,39	-0,28	-0,22	0,20	-0,24	0,30	-0,25	0,29	0,33	-0,28	0,20	-0,26	-0,29	0,25	-0,28	0,26	-0,30	-0,01
PC 36:4;O2	-0,42	0,29		0,97	0,65	-0,84	-0,89	-0,87	-0,70	0,71	-0,95	-0,42	0,86	-0,89	0,96	-0,91	0,84	0,94	-0,93	0,89	-0,90	-0,94	0,86	-0,94	0,89	-0,90	-0,43
PC 20:4;O/16:0	-0,42	0,30	0,97		0,67	-0,82	-0,88	-0,86	-0,67	0,74	-0,95	-0,40	0,89	-0,88	0,98	-0,91	0,87	0,97	-0,94	0,89	-0,90	-0,95	0,86	-0,95	0,90	-0,89	-0,49
PE 20:3;O/18:0	-0,43	0,12	0,65	0,67		-0,57	-0,61	-0,61	-0,31	0,63	-0,69	-0,31	0,68	-0,63	0,64	-0,65	0,62	0,65	-0,68	0,66	-0,64	-0,68	0,56	-0,68	0,57	-0,63	-0,20
PE 20:2/PGE1	0,44	-0,31	-0,84	-0,82	-0,57		0,97	0,95	0,63	-0,69	0,88	0,32	-0,54	0,90	-0,84	0,92	-0,79	-0,81	0,86	-0,74	0,91	0,93	-0,75	0,93	-0,81	0,96	0,34
PS 22:4/PGE1	0,45	-0,31	-0,89	-0,88	-0,61	0,97		0,97	0,65	-0,73	0,92	0,35	-0,64	0,92	-0,88	0,95	-0,84	-0,87	0,90	-0,79	0,93	0,96	-0,80	0,96	-0,86	0,98	0,37
PE 20:2/PGE1	0,45	-0,25	-0,87	-0,86	-0,61	0,95	0,97		0,62	-0,70	0,90	0,33	-0,65	0,91	-0,87	0,95	-0,81	-0,83	0,89	-0,78	0,91	0,95	-0,76	0,95	-0,83	0,97	0,34
PGD1	0,29	-0,20	-0,70	-0,67	-0,31	0,63	0,65	0,62		-0,48	0,67	0,24	-0,55	0,64	-0,69	0,65	-0,65	-0,67	0,65	-0,57	0,65	0,67	-0,59	0,67	-0,69	0,65	0,40
PG 40:5;O2	-0,55	0,39	0,71	0,74	0,63	-0,69	-0,73	-0,70	-0,48		-0,74	-0,23	0,63	-0,70	0,72	-0,73	0,67	0,74	-0,71	0,63	-0,72	-0,77	0,61	-0,77	0,71	-0,74	-0,12
PI 20:3;O/18:1	0,48	-0,28	-0,95	-0,95	-0,69	0,88	0,92	0,90	0,67	-0,74		0,38	-0,81	0,93	-0,94	0,96	-0,91	-0,94	0,99	-0,85	0,94	0,97	-0,89	0,98	-0,94	0,93	0,37
PS 18:0/20:4	0,22	-0,22	-0,42	-0,40	-0,31	0,32	0,35	0,33	0,24	-0,23	0,38		-0,37	0,34	-0,35	0,37	-0,39	-0,41	0,37	-0,30	0,36	0,38	-0,36	0,38	-0,39	0,34	0,02
PE 20:4;O/18:0	-0,35	0,20	0,86	0,89	0,68	-0,54	-0,64	-0,65	-0,55	0,63	-0,81	-0,37		-0,71	0,85	-0,73	0,71	0,84	-0,79	0,85	-0,72	-0,77	0,75	-0,77	0,76	-0,66	-0,39
PE 22:2/PGE1	0,47	-0,24	-0,89	-0,88	-0,63	0,90	0,92	0,91	0,64	-0,70	0,93	0,34	-0,71		-0,89	0,93	-0,79	-0,81	0,91	-0,77	0,98	0,95	-0,80	0,94	-0,86	0,92	0,35
PC 16:0/20:4;O	-0,43	0,30	0,96	0,98	0,64	-0,84	-0,88	-0,87	-0,69	0,72	-0,94	-0,35	0,85	-0,89		-0,91	0,85	0,95	-0,93	0,87	-0,90	-0,94	0,84	-0,94	0,87	-0,89	-0,53
PS 22:4/PGE1	0,45	-0,25	-0,91	-0,91	-0,65	0,92	0,95	0,95	0,65	-0,73	0,96	0,37	-0,73	0,93	-0,91		-0,85	-0,88	0,94	-0,82	0,95	0,97	-0,82	0,97	-0,88	0,96	0,35
PC 18:1;O/18:0	-0,46	0,29	0,84	0,87	0,62	-0,79	-0,84	-0,81	-0,65	0,67	-0,91	-0,39	0,71	-0,79	0,85	-0,85		0,90	-0,90	0,73	-0,81	-0,88	0,85	-0,89	0,91	-0,84	-0,39
PC 18:1;O/18:0	-0,44	0,33	0,94	0,97	0,65	-0,81	-0,87	-0,83	-0,67	0,74	-0,94	-0,41	0,84	-0,81	0,95	-0,88	0,90		-0,93	0,88	-0,83	-0,92	0,87	-0,92	0,90	-0,87	-0,50
3.20 901.5m/z	0,48	-0,28	-0,93	-0,94	-0,68	0,86	0,90	0,89	0,65	-0,71	0,99	0,37	-0,79	0,91	-0,93	0,94	-0,90	-0,93		-0,84	0,93	0,96	-0,87	0,96	-0,93	0,91	0,36
PC 20:4;O/18:0	-0,41	0,20	0,89	0,89	0,66	-0,74	-0,79	-0,78	-0,57	0,63	-0,85	-0,30	0,85	-0,77	0,87	-0,82	0,73	0,88	-0,84		-0,79	-0,86	0,76	-0,86	0,79	-0,81	-0,37
PE 18:1;O/20:0	0,45	-0,26	-0,90	-0,90	-0,64	0,91	0,93	0,91	0,65	-0,72	0,94	0,36	-0,72	0,98	-0,90	0,95	-0,81	-0,83	0,93	-0,79		0,96	-0,81	0,96	-0,88	0,94	0,35
PE 18:1;O2/18:0	0,48	-0,29	-0,94	-0,95	-0,68	0,93	0,96	0,95	0,67	-0,77	0,97	0,38	-0,77	0,95	-0,94	0,97	-0,88	-0,92	0,96	-0,86	0,96		-0,85	1,00	-0,91	0,97	0,37
PC 18:1;O/16:0	-0,47	0,25	0,86	0,86	0,56	-0,75	-0,80	-0,76	-0,59	0,61	-0,89	-0,36	0,75	-0,80	0,84	-0,82	0,85	0,87	-0,87	0,76	-0,81	-0,85		-0,86	0,90	-0,80	-0,47
PE 18:1;O/18:0	0,49	-0,28	-0,94	-0,95	-0,68	0,93	0,96	0,95	0,67	-0,77	0,98	0,38	-0,77	0,94	-0,94	0,97	-0,89	-0,92	0,96	-0,86	0,96	1,00	-0,86		-0,92	0,97	0,37
PC 18:1;O/16:0	-0,40	0,26	0,89	0,90	0,57	-0,81	-0,86	-0,83	-0,69	0,71	-0,94	-0,39	0,76	-0,86	0,87	-0,88	0,91	0,90	-0,93	0,79	-0,88	-0,91	0,90	-0,92		-0,86	-0,37
PC 18:1;O/16:0	0,49	-0,30	-0,90	-0,89	-0,63	0,96	0,98	0,97	0,65	-0,74	0,93	0,34	-0,66	0,92	-0,89	0,96	-0,84	-0,87	0,91	-0,81	0,94	0,97	-0,80	0,97	-0,86		0,35
PGD2	-0,06	-0,01	-0,43	-0,49	-0,20	0,34	0,37	0,34	0,40	-0,12	0,37	0,02	-0,39	0,35	-0,53	0,35	-0,39	-0,50	0,36	-0,37	0,35	0,37	-0,47	0,37	-0,37	0,35	

Significant correlation ($p<0.05$) is displayed in red. Coefficient values can be very small (0-0.9), small (0.1-0.29), moderate (0.3-0.49), large (0.5-0.69), very large (0.7-0.8) and nearly perfect (0.9-0.99) and perfect (1.00)
